# Determination of Levetiracetam in Human Plasma by Dispersive Liquid-Liquid Microextraction Followed by Gas Chromatography-Mass Spectrometry

**DOI:** 10.1155/2016/5976324

**Published:** 2016-10-17

**Authors:** Greyce Kelly Steinhorst Alcantara, Leandro Augusto Calixto, Luiz Alberto Beraldo de Moraes, Regina Helena Costa Queiroz, Anderson Rodrigo Moraes de Oliveira, Cristiane Masetto de Gaitani

**Affiliations:** ^1^Department of Pharmaceutical Sciences, Faculty of Pharmaceutical Sciences of Ribeirão Preto, University of São Paulo, 14040-903 Ribeirão Preto, SP, Brazil; ^2^Department of Exact and Earth Sciences, Institute of Environmental, Chemical and Pharmaceutical Sciences, Federal University of São Paulo, 09972-270 Diadema, SP, Brazil; ^3^Departament of Chemistry, Faculty of Philosophy, Sciences and Letters of Ribeirão Preto, University of São Paulo, 14040-901 Ribeirão Preto, SP, Brazil; ^4^Department of Clinical Analysis, Toxicology and Food Science, Faculty of Pharmaceutical Sciences of Ribeirão Preto, University of São Paulo, 14040-903 Ribeirão Preto, SP, Brazil

## Abstract

Levetiracetam (LEV) is an antiepileptic drug that is clinically effective in generalized and partial epilepsy syndromes. The use of this drug has been increasing in clinical practice and intra- or -interindividual variability has been exhibited for special population. For this reason, bioanalytical methods are required for drug monitoring in biological matrices. So this work presents a dispersive liquid-liquid microextraction method followed by gas chromatography-mass spectrometry (DLLME-GC-MS) for LEV quantification in human plasma. However, due to the matrix complexity a previous purification step is required. Unlike other pretreatment techniques presented in the literature, for the first time, a procedure employing ultrafiltration tubes Amicon® (10 kDa porous size) without organic solvent consumption was developed. GC-MS analyses were carried out using a linear temperature program, capillary fused silica column, and helium as the carrier gas. DLLME optimized parameters were type and volume of extraction and dispersing solvents, salt addition, and vortex agitation time. Under chosen parameters (extraction solvent: chloroform, 130 *μ*L; dispersing solvent: isopropyl alcohol, 400 *μ*L; no salt addition and no vortex agitation time), the method was completely validated and all parameters were in agreement with the literature recommendations. LEV was quantified in patient's plasma sample using less than 550 *μ*L of organic solvent.

## 1. Introduction

**Figure 1:**
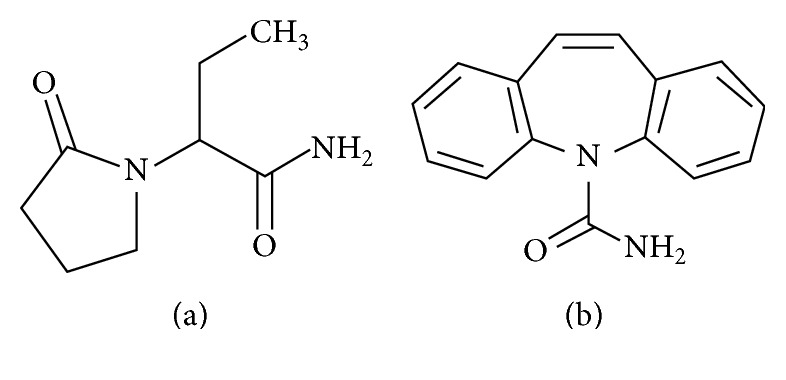
Chemical structures: (a) levetiracetam and (b) carbamazepine (IS).

Levetiracetam (LEV; Figure 1(a)) belongs to a generation of antiepileptic drugs that have been recommended for the treatment of epilepsy as either monotherapy in the case of partial seizures or as an adjunctive therapy for partial, myoclonic, and tonic-clonic seizures [1–3]. In addition, LEV exhibits benefits for other neurologic and psychiatric disorders, such as autism, anxiety, and bipolar disorders [1]. A unique pharmacokinetic profile [4–6] and multiple mechanisms of action have differentiated LEV from other antiepileptic drugs [3, 7]. According to the Subcommittee of the International League Against Epilepsy, the therapeutic plasmatic concentration of LEV was set from 12 to 46 mg L^−1^ [5, 8]. However, concentrations of LEV outside this range can be measured in special groups, such as elderly people, pediatric populations, pregnant patients, and patients with renal impairment. These situations could modify the expected plasma concentration, which may lead to a higher and toxic level of LEV [5].

Chromatographic methods for the quantification of LEV in biological matrices have been reported. These methods include high-performance liquid chromatography (HPLC) and gas chromatography (GC) with various detection systems [9–24]. Moreover, previously reported methods are mainly based on the conventional sample preparation procedure such as liquid-liquid extraction (LLE), solid-phase extraction (SPE), and plasmatic protein precipitation (PPP) [9–23]. The PPP procedure is considered a fast and easy method to remove the plasmatic protein; however, this procedure can result in signal suppression when liquid chromatography coupled to mass spectrometry (LC/MS) is used due to the presence of a precipitation agent, the coprecipitation of interfering species, and a lack of selectivity. In addition, the target analyte(s) may be occluded in the protein pellets [25]. The LLE procedure usually requires more consumption of hazardous solvents, is tedious, and involves multiple steps [26, 27]. Conversely, SPE is a more selective technique, so the ionization suppression is seldom observed. On the other hand, the cartridges used for the extraction are relatively expensive. Additionally, carryover or cross-contamination can occur [27].

Since 90s, analytical chemists have focused on the development of the new novel sample preparation techniques, highlighting the microextraction procedures. In general, the microextractions require low consumption of sample and organic solvents; besides they present adequate selectivity, high preenrichment, and suitable cleanup procedure.

Introduced in 2006 by Rezaee and collaborators, DLLME has received special attention in the analytical chemical field. It is a miniaturized kind of LLE which requires microliters of extraction solvent [28]. The principle consists of a ternary solvent system composed of sample (aqueous phase), extraction solvent, and dispersing solvent. The mixture of the extraction and dispersing solvent is quickly injected by microsyringe into the sample, containing the target analyte, creating a cloudy state due to the microdroplets of the extraction solvent dispersed into the aqueous phase. At this moment, a large surface area is formed between the fine droplets of the extraction solvent and the aqueous phase, the analyte transference toward the extraction phase occurring almost instantly [27–30]. After the centrifugation step, the extraction solvent microdroplets are sedimented at the bottom of the extraction tube and further collected by microsyringe and afterwards analyzed by an appropriate analytical technique [31].

This microextraction procedure has been widely developed to extract organic compounds from simple aqueous samples [30]. Nevertheless, there are not many works employing highly complex matrix [31]. For this purpose, this work will employ for the first time the DLLME to quantify an antiepileptic drug from human plasma by GC-MS.

## 2. Experimental

### 2.1. Chemicals, Reagents, and Standard Solutions

Standard stock solution of LEV purchased from Sigma Aldrich (St. Louis, MO, USA) was prepared at the concentration of 1 mg mL^−1^ in methanol. Appropriate dilutions were then made with methanol to obtain working stock solutions at concentrations range of 20-800 *μ*g mL^−1^. By spiking drug-free human plasma with the working solutions we obtained seven calibration standards (CS) and five levels of quality control (QC) defined by low (LQC), medium (MQC), and high (HQC) samples and the lowest limit (LLOQ) and upper limit (ULOQ) of quantification. The calibration curve was prepared at the concentrations of 2.0 (LLOQ), 4.0 (LQC), 10.0, 20.0, 40.0 (MQC), 60.0 (HQC), and 80.0 (ULOQ) *μ*g mL^−1^ in human plasma. A solution of carbamazepine (CBZ; Figure 1(b)) at the concentration of 100 *μ*g mL^−1^ was selected as internal standard (IS) (Sigma Aldrich, St. Louis, MO, USA). All these solutions were stored at −20°C in glass tubes.

The reagents (analytical grade) were dichloromethane and acetone, both acquired from Macron Chemicals (Philipsburg, NJ, USA). Acetonitrile and chloroethylene were purchased from Synth (Diadema, SP, Brazil). Methanol, carbon tetrachloride, and isopropyl alcohol were acquired from JT Baker (Philipsburg, NJ, USA). Chloroform was acquired from Tedia (Fairfield, CT, USA) and sodium chloride was purchased from Merck (Darmstadt, Germany). Water used to prepare the solutions was purified using a Milli-Q Plus System (Millipore, Bedford, USA).

### 2.2. GC System and Analytical Conditions

The GC-MS system used during the analyses was composed of a GC-2010 plus Gas Chromatograph coupled to a mass spectrometry model QP2010 Series Plus system equipped with an autosampler model AOC20i, with electron impact (70 eV) as the ionization source, all of which were obtained from Shimadzu Technologies (Kyoto, Japan). The chromatographic separations were made using Rtx-5ms (5% phenyl/95% dimethyl polysiloxane, 30 m length × 0.25 mm* i.d, *0.25 *μ*m film thickness) fused silica capillary columns from SGE Analytical Science (Ringwood, Australia). The injection and ionization source temperatures were 250°C and 220°C. The initial temperature of the column oven was 150°C. The column temperature was then programmed to increase to 230°C at a rate of 20°C min^−1^, at which point it was held for 2 min before finally being increased by 20°C min^−1^ to 270°C (and again held for 1 min). The column flow rate using pure helium (99.999%) was set at 1 mL min^−1^, and the total analytical time was 9 min. Simultaneously, the full scan mode was obtained over a mass range from* m/z* 40 to 400 to confirm the identification of the analytes. Quantification of LEV and the IS was carried out in selected ion monitoring (SIM) mode at the following mass/charge (*m/z*) ratios:* m/z* 123 for LEV and* m/z* 193 for the IS. LabSolution 2.53 software from Shimadzu was used to control the GC-MS system and for data acquisition.

### 2.3. Plasma Samples

The Ethics Committee of the College of Pharmaceutical Sciences from Ribeirão Preto, University of São Paulo (protocol number 898.318), approved the protocol for this study. The Hemotherapy Center of Ribeirão Preto donated drug-free plasma samples from healthy volunteers. A patient treated with LEV received all information about the study protocol and gave written informed consent to participate in the research. Blood samples of patients treated with LEV (±5 mL) were collected in a Vacutainer heparinized tube (Becton Dickinson, Meylan, France) and centrifuged at 1800 ×g for 5 min. After separation of the plasma sample, it was stored in a polypropylene tube and kept frozen at −20°C until the analysis.

### 2.4. Sample Preparation

#### 2.4.1. Ultrafiltration Procedure

In conical bottom glass tubes (*n* = 4) 500 *μ*L of human plasma sample, 50 *μ*L of the LEV solution (400 *μ*g mL^−1^), 50 *μ*L of the IS solution (100 *μ*g mL^−1^), and 1.0 mL of ultrapure water were added. The tubes were vortex for 1 minute and this solution was transferred to a 15 mL Amicon ultrafiltration tube with a membrane of 10 kDa porous size from Millipore® (Darmstadt, Germany). These ultrafiltration tubes were centrifuged at 1800 ×g for 20 minutes using a CF-15 centrifuge (Hitachi Koki, Kyoto, Japan). Then, 1.0 mL of the permeated phase (aqueous solution containing LEV and IS) was transferred to a conical bottom glass tube for further DLLME optimization procedures. So the Amicon ultrafiltration tubes were reused among the analysis. In order to assess the carryover effect, an aliquot of 500 *μ*L of human plasma sample and 1100 *μ*L of ultrapure water were added in Amicon ultrafiltration tubes, after DLLME extraction of ULOQ solution, and centrifuged at 1800 ×g for 20 minutes (blank plasma samples). The permeated phase was subjected to DLLME procedure. The washing procedure of Amicon ultrafiltration tubes consisted of the use of ultrapure water at least four times.

#### 2.4.2. DLLME

**Figure 2:**
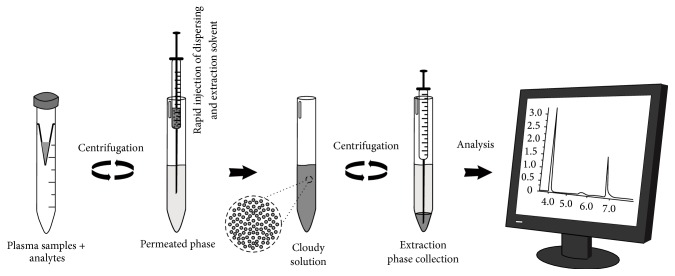
Steps of DLLME procedure.

For DLLME procedure, an aliquot of 1.0 mL of pretreated plasma (described in Section 2.4.1) samples was added in a 10 mL conical bottom glass tube. 400 *μ*L of isopropyl alcohol (dispersing solvent) containing 130 *μ*L of chloroform (extraction solvent) was injected rapidly into the aqueous phase with a 1.0 mL microsyringe (Gastight, Hamilton, Reno, NV, USA). At the same moment, a cloudy solution (aqueous phase/extraction solvent/dispersing solvent) was quickly formed in the conical tube. So the conical tube was subjected to centrifugation at 1800 ×g for 5 minutes. After centrifugation, fine droplets of the extraction solvent were concentrated in the bottom of the tube (sedimented phase). The volume of the sedimented phase (*μ*L) was determined, and the solution was transferred to a clean tube for solvent evaporation under a gentle stream of compressed air. The residue was solubilized in 200 *μ*L of methanol, and 1 *μ*L of the sample was injected into the GC-MS system. The procedure is summarized in Figure 2.

All optimization parameters, such as the type of extraction and dispersing solvents, the volume of extraction and dispersing solvents, the ionic strength, and the vortex agitation time (assisted DLLME), were applied in quadruplicate experiments (*n* = 4). The extraction efficiency was determined by plotting the peak area of LEV* versus* the evaluated parameter. The results of each parameter were analyzed using the Minitab 14.0 statistical program (State College, PA, USA).

### 2.5. Validation Method

Validation method was carried out according to the EMA guidelines on bioanalytical method validation [45]. The method linearity was evaluated by employing seven different concentrations of LEV in quadruplicate (*n* = 4), and the results were weighted by 1/*x*. A calibration curve was obtained by spiking 500 *μ*L of human plasma with 50 *μ*L of the standard solution of LEV, 50 *μ*L of the IS, and 1.0 mL of ultrapure water. This solution was submitted to ultrafiltration procedure (described in Section 2.4.1) and then DLLME procedure (described in Section 2.4.2). The calibration curve range was from 2 to 80 *μ*g mL^−1^. Linear regression was performed by plotting the peak area ratios between LEV and the IS* versus* the LEV concentrations. After that, the statistical parameters of the calibration curve were calculated by analysis of variance (ANOVA) and linear regression [32, 46].

The lower limit of quantification (LLOQ) of the method was also evaluated and it was defined as the lowest concentration that can be quantified reliably with an accuracy and precision below 20% of a nominal value [45].

Within-day accuracy and precision were evaluated using five replicates (*n* = 5), in which 500 *μ*L of plasma samples was spiked with a minimum of four concentration levels covering the calibration curve range (LLOQ, LQC, MQC, and HQC) of the standard solution of LEV. Between-day precision and accuracy were assessed for at least three consecutive days of operation. The precision and accuracy parameters were expressed as relative standard deviation (RSD, %) and as percentage of relative error (RE, %), respectively [45].

The selectivity was assessed by analyzing six individual sources of the blank matrix (drug-free human plasma), which included four normal plasma sources, a hemolyzed plasma source, and a hyperlipidemic plasma source in order to verify that no endogenous peak would interfere in the LEV and the IS signals under the GC-MS conditions previously established [45].

The carryover parameter was assessed by injecting blank plasma samples (without the standard LEV spike) after injecting the calibration LEV standard at the upper limit of quantification (80 *μ*g mL^−1^). According to the EMA (2011), the carryover effect on the blank sample should not be greater than 20% of the LOQ and 5% for the IS [45].

The stability parameter was determined in order to ensure that the concentration of LEV had not been affected by the storage conditions to which the analyte was subjected. Therefore, the following stability tests were performed: (i) freeze (−20°C)/thaw (22 ± 2°C) cycles, (ii) short-term room temperature conditions (8 h on the bench-top), and (iii) autoinjector conditions (24 hours, 22 ± 2°C). To conduct the stability tests, 500 *μ*L of human plasma was spiked with LEV at a LQC (4.0 *μ*g mL^−1^) and at a HQC (60.0 *μ*g mL^−1^). After submitting samples at storage and preparation conditions, the IS was added and developed ultrafiltration and DLLME procedures with the replicates. The LEV concentrations obtained from the stability tests were compared with the LEV concentrations obtained from freshly prepared samples (*n* = 4). The samples were considered stable if the mean concentrations at each concentration were within ±15% of the nominal concentration value [45].

## 3. Results and Discussion

### 3.1. Chromatographic Separation

To date, no method has been described in the literature for the quantification of LEV from human plasma by GC-MS. One of the aims of this work was to develop a sensitive, selective, fast, and accurate methodology for plasma LEV analysis employing the DLLME as sample preparation technique. Based on that goal, a GC system with electron impact as the ionization source and an Rtx-5ms fused silica capillary column was employed. The IS chosen for analysis was CBZ solution prepared at 100 *μ*g mL^−1^ in methanol. The initial oven temperature was 150°C, and then it was increased by 20°C/min to 230°C (held for 2 min) and reached a final temperature of 270°C (held for 1 min). Under these chromatographic conditions, the retention times were 4.2 min and 7.2 min for LEV and the IS, respectively. However, several ramping speeds and temperatures were evaluated in order to obtain a GC run-time that was as short as possible.

Initially, the SIM-mode chosen for the quantification of single ions was* m/z* 126 for LEV and* m/*z 193 for the IS. During the selectivity evaluation, a blank sample was injected and the peaks from endogenous compounds of human plasma sample were eluted at the same time of LEV (4.2 min) using SIM-mode* m/z* 126. Based on this result, another single ion* m/z* 123 was selected for further analysis of LEV, and no peaks from endogenous compounds were observed at the retention time of LEV or the IS.

### 3.2. Ultrafiltration Procedure

DLLME procedure is a technique widely employed to extract analytes from environmental samples but it is seldom applied for the extraction of analytes from biological samples. Endogenous compounds from a biological matrix could interact with organic solvents, and a suitable sedimented phase could not be formed [28]. Some strategies have been adopted to remove the influence of this matrix during the DLLME procedure. Mashayekhi et al. [32] reported the DLLME procedure for the quantification of CBZ where a suitable amount of acetonitrile was employed to reduce the matrix effect and then centrifuged was done. After filtering, dilution was done ten times for the DLLME procedure. In the same way, Rezaee et al. [25] carried out the determination of letrozole in human plasma. Adopted different approaches of pretreatment as well as DLLME application in biological matrices were showed in Table 1. Different from the previous published papers, this work presents for the first time the use of ultrafiltration procedure as previous treatment of human plasma of DLLME.

Ultrafilters were successfully applied to reduce the matrix effect of the plasma samples. A limpid aqueous phase, nominated permeated phase, was obtained. Therefore, a new pretreatment for complex matrix by DLLME was introduced, without adding organic solvent, resulting in less waste and, consequently, diminishing the environmental concern.

So 500 *μ*L aliquot of the plasma sample, which was previously spiked with the LEV standard solution (400 *μ*g mL^−1^) and the IS solution (100 *μ*g mL^−1^), and 1.0 mL of ultrapure water were placed into Amicon ultrafiltration tubes and centrifuged at 1800 ×g for 20 minutes. Afterwards, 1.0 mL of the permeated phase, containing LEV and IS, was transferred to a conical bottom glass tube for further DLLME optimization. Membrane of the Amicon ultrafiltration tubes was porous with 10 kDa size which it enables it to block the plasmatic proteins and permit the passage of the LEV and IS due to low size of molecules, 170 and 236 daltons, respectively.

The Amicon ultrafiltration tubes were reused during developing of the method. The washing procedure consisted of the use of ultrapure water at least four times to remove the plasma proteins. Further, the method assessed the carryover effect of the membranes of Amicon ultrafiltration tubes. So a blank plasma sample (without LEV and IS) was processed in ultrafiltration tubes previously used with ULOQ of LEV, in quadruplicate. There was no carryover effect among the analysis. So the washing procedure was suitable, once precision and accuracy values were in agreement with the specification of the regulatory agency.

### 3.3. DLLME

LEV is an antiepileptic drug novel with polar nature and it is fairly challenging for DLLME technique. LEV presents a significant tendency to remain in the aqueous phase once it has low octanol-water partition coefficient [33]. Nevertheless, the drug shows that it is freely soluble in chloroform and methanol, soluble in ethanol, sparingly soluble in acetonitrile, and practically insoluble in n-hexane [4]. However, there is possibility to extract the drug from aqueous phase.

In order to reach the best extraction efficiency of LEV, type and volume of the extraction and dispersing solvents, ionic strength, and the vortex agitation time (assisted DLLME) were optimized. All of these DLLME parameters evaluated were carried out in quadruplicate (*n* = 4). The applicability of the developed method was confirmed by checking a plasma sample from a patient who had daily taken LEV.

#### 3.3.1. Selection of Extraction Solvent

**Figure 3:**
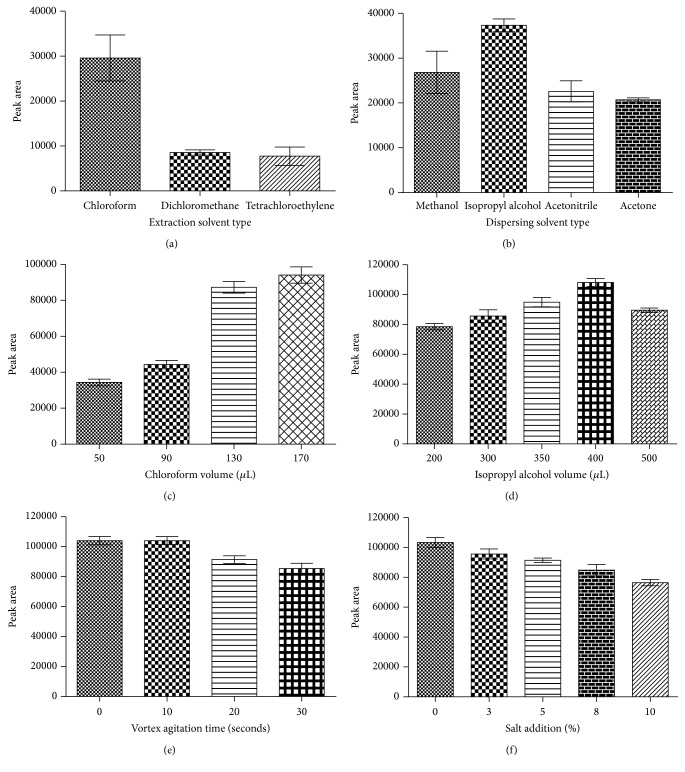
Optimization of DLLME procedure: (a) extraction solvent type, (b) dispersing solvent type, (c) extraction solvent (chloroform) volume, (d) dispersing solvent (isopropyl alcohol) volume, (e) vortex agitation time, and (f) salt addition.

The selection of the extraction solvent type is a fundamental step in the DLLME procedure [32]. During this step, it is highly important to choose solvents with a higher density than water and a low solubility in water that shows good chromatographic behavior and extraction capability for the analyte of interest [34, 35]. Chlorinated solvents, such as dichloromethane, tetrachloroethylene, carbon tetrachloride, and chloroform, show these characteristics. The effect of these solvents on LEV extraction was observed by using a fixed volume of 210 *μ*L of methanol (dispersing solvent) combined with 90 *μ*L of each chlorinated solvent. This mixture was injected directly by syringe into 1.0 mL of the permeated phase containing the analyte of interest and the IS. During these experiments, a low volume of the sedimented phase was observed when carbon tetrachloride was used as the extraction solvent, which prevents this sample from being further collected and analyzed. Chloroform showed higher extraction efficiency probably because LEV is highly soluble in chloroform (Figure 3(a)). Therefore, chloroform was chosen as the extraction solvent for the subsequent DLLME optimization.

#### 3.3.2. Selection of Dispersing Solvent

The most important point for the choice of a dispersing solvent is its miscibility in both aqueous phase and organic phase [25]. Thereby, 210 *μ*L of each selected dispersing solvent, methanol, acetonitrile, acetone, and isopropyl alcohol, was evaluated mixing 90 *μ*L of chloroform. Excluding isopropyl alcohol, all other dispersing solvents demonstrated similar extraction efficiency for LEV in human plasma (Figure 3(b)). Isopropyl alcohol assisted in the dispersion of the extraction solvent inside aqueous phase, leading a formation of an adequate cloudy state [47–49]. However, it was selected for further experiments once it showed the best efficiency of extraction of LEV.

#### 3.3.3. Effect of Extraction Solvent Volume

The influence of the volume of chloroform was evaluated in the following range while keeping the volume of isopropyl alcohol constant (210 *μ*L): 50, 90, 130, and 170 *μ*L. After the addition of 50 *μ*L of chloroform into the aqueous phase, a very low amount of sedimented phase was formed. Due to the low volume of the sedimented phase, the reproducibility was poor, making it impossible to use this amount of extraction solvent for quantitative analysis. The difficulty in collecting low amounts of the sedimented phase has been previously reported by López-Nogueroles et al. [48]. Nevertheless, by adding 130 *μ*L and 170 *μ*L of chloroform, the area peaks associated with LEV were compared using analysis of variance (ANOVA). There was no statistically significant difference considering a confidence level of 0.05. Therefore, 130 *μ*L of chloroform was chosen to perform the DLLME procedure (Figure 3(c)).

#### 3.3.4. Effect of Dispersing Solvent Volume

To verify the effect of dispersing solvent volumes was investigated by mixing 130 *μ*L of chloroform with 200, 300, 350, 400, and 500 *μ*L of the isopropyl alcohol and then rapidly injecting the samples into 1.0 mL of the aqueous phase. The results demonstrated that the extraction efficiency of LEV slightly increased after the addition of isopropyl alcohol in amounts from 200 to 350 *μ*L. The maximum value was reached when adding 400 *μ*L of isopropyl alcohol (Figure 3(d)). Probably at lower volumes of isopropyl alcohol (less than 400 *μ*L), the cloudy state did not form very well, and thus, the extraction efficiency of LEV decreased. Furthermore, it seems that by further increasing the isopropyl alcohol volume (more than 400 *μ*L), no statistically significant difference was observed by ANOVA (*P* > 0.05). Therefore, 400 *μ*L of isopropyl alcohol was selected for further optimization.

#### 3.3.5. Effect of Vortex Agitation Time

In addition to the features of other microextraction techniques, DLLME has the advantage of saving time [29]. This is due to the extremely large surface area between the aqueous and extraction phases; therefore, the equilibrium state can be achieved quickly (a few seconds), and the analyte transfer is almost immediate from the aqueous phase to the extraction phase [25, 28, 30, 47]. To increase the extraction efficiency of LEV, vortex-mixing after the formation of the cloudy mixture has been proposed (assisted DLLME). The agitation time varied from 10 to 30 seconds after the cloudy state formation. The results showed no statistically significant difference (ANOVA, *P* > 0.05) when compared with the tubes without vortex agitation (Figure 3(e)). Therefore, the extraction time was defined as the time after injecting the mixture of extraction/dispersing solvent into the aqueous phase until centrifugation without vortex-mixing.

#### 3.3.6. Salt Addition

In the DLLME procedure, the salting-out effect was assessed by adding NaCl ranging from 3 to 10% (*w/v*) to the aqueous phase in order to increase the extraction efficiency of LEV from human plasma (Figure 3(f)). Water molecules form hydrated spheres around the salt ions, thus reducing the water quantity available to dissolve the target analyte and thereby leading the analyte molecules to the extraction solvent [36, 50]. However, in competition with this process, polar molecules such as LEV can participate in electrostatic interactions with the salt ions in solution and can consequently reduce the ability of the analyte to transfer to the organic solvent droplets [36, 50]. As a result, the subsequent experiments were carried out without the addition of salt. Once the mixture reached a cloudy state (formation of a large surface area), the centrifugation step was a fundamental procedure to separate both the aqueous and the extraction systems. According to previous studies, centrifugation times greater than 5.0 minutes do not promote higher efficiencies of the extraction [47]. Therefore, 5 minutes and 1800 ×g were set as suitable conditions for achieving the separation of the sedimented phase and promoting good extraction efficiency. The final conditions for the DLLME procedure are summarized in Table 2.

### 3.4. Validation Method

Linearity of the analytical method was investigated by employing seven concentrations of LEV in four replicates. Thus, an aliquot of 500 *μ*L of human plasma samples spiked with 50 *μ*L of LEV and 50 *μ*L of IS in order to obtain the final concentrations of 2 (LLOQ), 4 (LQC), 10, 20, 40 (MQC), 60 (HQC), and 80 (ULOQ) *μ*g mL^−1^ of LEV and 10 *μ*g mL^−1^ of CBZ. So, 1.0 mL of ultrapure water was added and the conical glass tubes were submitted to vortex for 1 minute. The solution was transferred to Amicon ultrafiltration tubes and it was centrifuged at 1800 ×g for 20 minutes. Afterwards, DLLME (described in Section 2.4.2) procedure was developed in 1.0 mL of obtained permeated phase. The results were weighted (1/*x*) because the residual analysis of the analytical curve showed heteroscedasticity behavior. The curve calibration was linear over the concentration range from 2 to 80 *μ*g mL^−1^. The linear equation calculated by the least squares method was *y* = 0.0040*x* + 0.0011 (*r* = 0.9988; RE = 0.7%; RSD = 5.8%). The results are showed in Table 3. According to the AMC (Analytical Methods Committee) [51], a value of the regression coefficient (*r*) close to unity is not necessarily the outcome of a linear relationship. Thus, a test for the lack of fit should be performed [52]. This test evaluates the variance of the residual values for each concentration [53]. For lack of fit, the calculated *F* value (1.15) was smaller than the tabulated *F* value (2.84) and the *P* value was higher than 0.05 (95% significance level), whereas for the linear regression model, the calculated *F* value (4931.71) was higher than tabulated *F* value (2.85) and its *P* value was smaller than 0.05 (95% significance level), as determined by ANOVA. According to the statistical tests, the outcome of the analysis by the regression method is linear and showed no lack of fit (Table 4).

The within- and between-days precision and accuracy evaluated for at least four concentrations of the LEV standard, in quintuplicate, are given in Table 5. Then, the measured concentration at each concentration level was within ±15% of the nominal concentration and for the LLOQ within ±20%.

Stability tests assured the LEV stability in the biological matrix. Therefore, all tests performed, such as the freeze and thaw cycles, short-term room temperature conditions, and autoinjector conditions (Table 6) showed no significant instability.

## 4. Method Application and Comparison to Other Methods

The analytical parameters of the proposed method have been compared to earlier reported methods for the quantitation of LEV. Greiner-Sosanko et al. [21] described a GC-NPD method where the linear range was from 2.5 to 45 *μ*g mL^−1^. Our developed method presented a wider linear range compared to this previous method. The GC-MS method developed by Isoherranen et al. [9] showed a total run-time for the LEV of 14.7 min, which is longer time than our proposed run-time (9.0 min). Until this moment, the literature has no described method to quantify LEV from biological sample using a miniaturized sample preparation technique. In this regard, DLLME procedure was able to promote a suitable cleanup procedure of the biological samples and to extract the LEV despite its polar nature from aqueous phase employing only 130 *μ*L of chloroform. Previously to DLLME, the ultrafiltration procedure was employed for the first time to remove endogenous compounds from the plasma allowing accomplishment of the DLLME procedure, even in complex matrix. Unlike other pretreatments described in the literature for DLLME, this one does not require the use of any organic solvents. The ultrafilters were reused among analysis after checking carryover effect.

**Figure 4:**
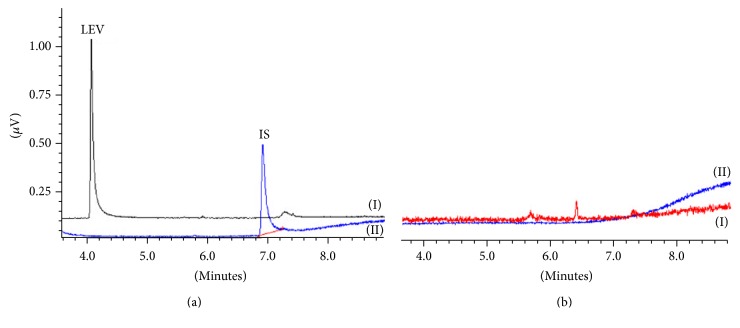
GC-MS chromatograms obtained from the analysis of an extracted patient plasma sample: (a) patient sample after the levetiracetam intake (plasma concentration of levetiracetam was 25 *μ*g mL^−1^) and (b) a drug-free plasma sample; SIM mode is (I) m/z 123 and (II) m/z 193.

The proposed analytical method was used to analyze a human plasma sample from a patient who had taken LEV (1000 mg once per day) in order to evaluate its applicability. Patient plasma samples and drug-free plasma were extracted under optimized conditions (*n* = 3), and their chromatograms are shown in Figure 4. The plasmatic concentration of LEV in the patient was 25 *μ*g mL^−1^ (RSD = 3.2% and RE = 5.6%), demonstrating it is within the therapeutic range. Therefore, the developed method has successfully been applied in the therapeutic monitoring of a patient taking LEV.

## 5. Concluding Remarks

For the first time, an analytical method has been described employing DLLME/GC-MS for the determination of LEV in human plasma. The chromatographic separation was very simple, fast, easy to operate, accurate, and precise. Additionally, this work has demonstrated an alternative cleanup pretreatment able to remove endogenous interferences from the plasma sample without the use of any organic solvents. During all DLLME-GC-MS presented here, low organic solvent consumption (*μ*L) has been required to analyze a complex matrix, and consequently the operational costs of the process have been reduced, as well as the low waste generation.

## Figures and Tables

**Table 1:** DLLME application in biological matrices and pretreatment approaches.

Analytes	Sample	Pretreatment	Extracting solvent volume (*µ*L)	Dispersing solvent volume (*µ*L)	Analysis method	References
Carbamazepine	Plasma	Plasma: precipitation with acetonitrile, centrifugation, filtration (0.45 *µ*m), and dilution (1 : 10)	Chloroform (78 *µ*L)	Ethanol (1000 *µ*L)	HPLC-UV/Vis	[32]
	Urine	Urine: dilution (1 : 5)				

Amitriptyline, clomipramine, and thioridazine	Urine	Centrifugation for 15 min and filtration (0.45 *µ*m) of upper phase	Carbon tetrachloride (20 *µ*L)	Acetonitrile (500 *µ*L)	HPLC-UV/Vis	[33]

Clozapine and chlorpromazine	Urine	Centrifugation at 4000 rpm/15 min	Carbon tetrachloride (40 *µ*L)	Ethanol (200 *µ*L)	HPLC-UV/Vis	[34]

Losartan and carvedilol	Plasma	Plasma: precipitation with acetone (1 : 1) and dilution (1 : 5)	Plasma: chloroform (100 *µ*L)	Plasma: acetone (500 *µ*L)	HPLC-UV/Vis	[35]
	Urine	Urine: pH adjustment (acid solution)	Urine: chloroform (160 *µ*L)	Urine: acetone (400 *µ*L)		

7-Aminoflunitrazepam	Urine	Ammonia (0.2 M)	Dichloromethane (250 *µ*L)	Isopropyl alcohol (500 *µ*L)	LC-ESI-MS/MS	[36]

Fentanyl, alfentanil, and sufentanil	Plasma	Plasma: filtration (0.45 *µ*m), dilution (1 : 4), and precipitation with methanol (1 : 2)	Chloroform (142 *µ*L)	Methanol (2000 *µ*L)	HPLC-UV/Vis	[37]
	Urine	Urine: filtration (0.45 *µ*m) and dilution (1 : 2)				

Piroxicam	Urine	Enzymatic conjugation	Chloroform (70 *µ*L)	Methanol (700 *µ*L)	Espectrofotometer	[38]

Methadone	Plasma Urine	Plasma and urine centrifugation, decantation, filtration, and dilution (1 : 20)	Chloroform (250 *µ*L)	Methanol (2500 *µ*L)	HPLC-UV/Vis	[39]
	Saliva	Saliva: centrifugation and dilution (1 : 100)				
	Sweat	Sweat: successive baths of ethanol (30 min each bath)				

Warfarin	Plasma	Precipitation with trichloroacetic acid (10%), refrigeration (4°C/20 min), and dilution	1-Octanol (150 *µ*L)	Methanol (150 *µ*L)	HPLC-UV/Vis	[40]

Opium alkaloids	Urine	Centrifugation, filtration, and dilution	Chloroform (88 *µ*L)	Acetone (1000 *µ*L)	HPLC-UV/Vis	[41]

Five antiarrhythmic drugs	Plasma	Precipitation with acetonitrile	Dichloromethane (100 *µ*L)	Acetonitrile (1340 *µ*L)	HPLC-UV/Vis	[42]

Benzodiazepines	Plasma	Precipitation with acetonitrile	Chloroform (220 *µ*L)	Acetonitrile (3200 *µ*L)	HPLC-UV/Vis	[43]

Efavirenz	Plasma	Sulfosalicylic acid (4%)	Chloroform (100 *µ*L)	Acetonitrile (1300 *µ*L)	HPLC-UV/Vis	[44]

**Table 2:** DLLME parameters.

Optimized parameters		Defined conditions
Dispersing solvent types	Methanol, isopropyl alcohol, acetonitrile, and acetone	Isopropyl alcohol
Extraction solvent types	Chloroform, dichloromethane, carbon tetrachloride, and tetrachloroethylene	Chloroform
Dispersing solvent volumes (*µ*L)	200, 300, 350, 400, and 500	400
Extraction solvent volumes (*µ*L)	50, 90, 130, and 170	130
NaCl concentration (%)	0–10	0
Type of shaking (seconds)	0–30	0

**Table 3:** Linearity and limit of quantification of the LEV method for analyses of human plasma.

Parameters	LEV
Slope^a^	0.0040
Intercept^b^	0.0011
Regression coefficient (*r*)	0.9988
Linear range (*µ*g mL^−1^)	2–80
Experimental *F* value^b^	1.15
*P* value	0.37

LLOQ (*µ*g mL^−1^)	2
Precision (RSD, %)	5.8
Accuracy (RE, %)	0.7

^a^Calibration curves were prepared in quadruplicate (*n* = 4) for concentrations of 2, 4, 10, 20, 40, 60, and 80 *µ*g mL^−1^; *y* = *Ax* + *B*, where *y* is the ratio between the analyte peak area and the IS peak area; *A* is the slope, *B* is the intercept, and *x* is the concentration of the measured solution in *µ*g mL^−1^; ^b^experimental *F*_value_ < *F*_crit,95%_ = 2.84.

**Table 4:** ANOVA results for the linearity of LEV (SS: sum of squares; Df: degrees of freedom; MS: mean squares; *F*_calc_: calculated *F* test; *F*_tab_: tabulated *F* test).

ANOVA	SS	Df	MS	*F*_calc_	*P* value^a^	*F*_tab_
Regression model	0.26854	1	0.26854	4931.71	0.000	2.85
Residual error	0.00103	19	0.00005	Linear		
Lack of fit	0.00030	5	0.00006	1.15	0.379	2.84
Pure error	0.00073	14	0.00005	No lack of fit		

^a^ *P* value: significance level of 0.05.

**Table 5:** Within- and between-days accuracy and precision.

Analyte	Nominal concentration (*µ*g mL^−1^)	Measured concentration (*µ*g mL^−1^)	Precision RSD^a^ (%)	Accuracy RE^a^ (%)
Within-day				
LEV	2.0	1.9	10.6	−3.1
	4.0	4.1	11.4	3.1
	40.0	38.0	7.6	−5.0
	60.0	57.8	8.9	−3.6

Between-day				
LEV	2.0	2.0	4.6	1.6
	4.0	4.2	−8.1	5.1
	40.0	39.1	3.0	−2.4
	60.0	61.6	4.0	2.6

^a^RSD, relative error deviation expressed as percentage (%).

^b^RE, relative error expressed as percentage (%).

**Table 6:** Stability assays (*n* = 4) of the developed method.

Nominal concentration (*µ*g mL^−1^)	Short-term room temperature (8 h)		Autoinjector (24 h)		Freeze and thaw cycles (12 h, 3 cycles)	
	Accuracy RE^a^ (%)	Precision RSD^b^ (%)	Accuracy RE^a^ (%)	Precision RSD^b^ (%)	Accuracy RE^a^ (%)	Precision RSD^b^ (%)
4.0	2.3	2.4	4.2	2.5	1.6	3.2
60	5.2	4.3	5.6	4.2	5.8	4.1

^a^RE, relative error expressed as percentage (%).

^b^RSD, relative error deviation expressed as percentage (%).
